# Importance of Video-EEG Monitoring in the Diagnosis of Refractory Panic Attacks

**DOI:** 10.1155/2013/340792

**Published:** 2013-07-24

**Authors:** Batool F. Kirmani, Diana Mungall

**Affiliations:** ^1^Epilepsy Center, Department of Neurology, Scott & White Neuroscience Institute, TX 76508, USA; ^2^Texas A&M Health Science Center College of Medicine, Temple, TX, USA

## Abstract

Partial seizures can be misdiagnosed as panic attacks. There is considerable overlap of symptoms between temporal lobe seizures and panic attacks making the diagnosis extremely challenging. Temporal lobe seizures can present with intense fear and autonomic symptoms which are also seen in panic disorders. This results in delay in diagnosis and management. We report an interesting case of a young woman who was diagnosed with right temporal lobe seizures with symptoms suggestive of a panic attack.

We report an interesting case of a 24-year-old woman who sustained a motor vehicle accident at the age of sixteen resulting in significant head trauma. She was an unrestrained passenger and suffered major injuries, including an epidural hematoma. She was hospitalized and was in a coma for 3.5 months. She underwent extensive speech and physical therapy after the incident. She had residual mild cognitive decline and developed episodes characterized by anxiety, fear, whole body tingling, and associated autonomic symptoms lasting between one and two minutes. Interestingly, there was preservation of consciousness and speech during these episodes. She is able to carry on a normal conversation during and after the event. She does have a psychiatric history which includes depression and prolonged periods of irritability resulting in verbal outbursts even before the accident. She was admitted to the psychiatry facility a few years ago as a result of a hypomanic episode. Given her history, panic attacks were still high on the differential diagnosis. She was referred to an outside neurologist based on her history of head trauma and refractory nature of the episodes; seizures were considered in the differential diagnosis because of head trauma and abnormal imaging. Her electroencephalogram (EEG) was negative but magnetic resonance imaging did show encephalomalacia in the right temporal lobe. She was given a trial of valproic acid which did not affect the frequency of spells. She was eventually switched to a low dose of Lamotrigine XR 100 mg daily for mood stabilization and 4 possible seizures. She continues to have these episodes with a frequency of one to two per week. She was referred to our epilepsy center eight years after head trauma because of refractory panic attacks and suspicion of seizures due to head trauma. She was admitted to the inpatient unit for intensive video-EEG monitoring to capture these spells for definitive diagnosis. We were able to capture a few of her stereotypical episodes and they did correlate with abnormal brain waves. EEG revealed seizure activity in the right temporal region during these episodes (Figure 1). She was diagnosed with partial epilepsy in the right temporal region and anticonvulsant medications were adjusted which resulted in improvement in her condition. The dose of Lamotrigine XR was increased to 200 mg daily. Levetiracetam extended release 1000 mg daily was added and vagal nerve stimulator was implanted few months later which resulted in cessation of seizures.

This report emphasizes the need for urgent referral to a specialized epilepsy center for adequate management if there is suspicion of partial seizures. In our patient, the diagnosis seemed to be challenging because of the past psychiatry history and atypical presentation of the episodes which favors panic attacks over seizures. She had preservation of consciousness and speech during her episodes which lasted between one and two minutes. The right temporal seizure focus explains the preservation of speech which shows left cerebral hemispheric dominance. She was given a trial of anticonvulsants but always remained on suboptimal doses based on the suspicion that these were panic attacks and not focal seizures. Not much therapeutic benefit was achieved over all these years since a definitive diagnosis was not established. 

Partial seizures are included in the differential diagnosis of panic disorders but diagnosis remains extremely challenging. It has been shown in the literature that lifetime prevalence of epilepsy is 3-4% and about 60% of those have complex partial seizures [1, 2]. The prevalence of panic attacks is about 1.5% [3].

There is also considerable overlap between the symptoms of the two disorders [4]. Panic disorder may present with derealization, depersonalization, and fear of losing control or dying, which are classified under affective symptoms. The physical symptoms manifested during panic attacks include nausea, chills, paraesthesias, choking feeling, faintness, palpitations, chest pains, trembling, and breathlessness. According to the Diagnostic and Statistical Manual of Mental Disorders, fourth edition, the patient should have at least four of these symptoms [5]. The temporal lobe seizures are also characterized by fear and autonomic symptoms making the diagnosis difficult based on the clinical history alone [6].

Anticonvulsants mask the EEG findings. Furthermore, routine EEGs may appear normal or may show nonspecific focal abnormalities, further complicating the diagnosis. The diagnosis of partial epilepsy should not be excluded based on normal EEG and prolonged inpatient video-EEG monitoring under medication withdrawal for 3–5 days should be considered a gold standard so that the spells can be captured for definitive diagnosis [7, 8]. 

## Figures and Tables

**Figure 1:**
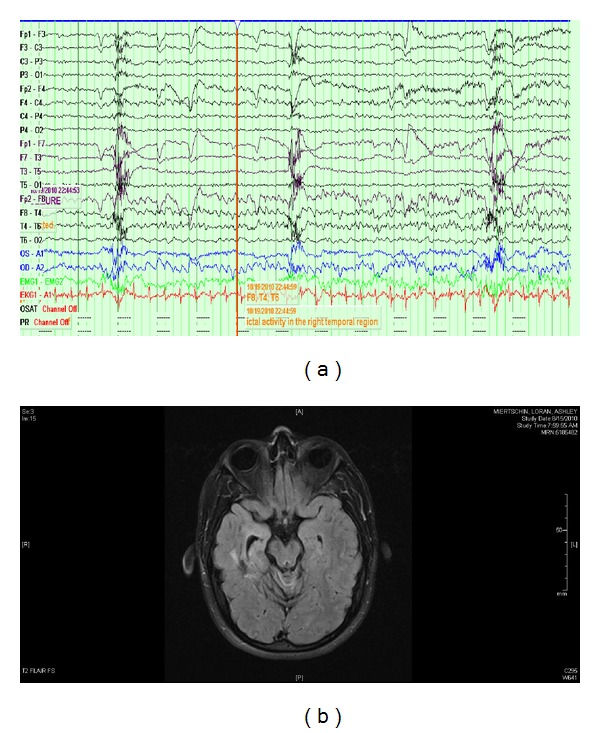
(a) Electrographic seizure in the right temporal region (F8, T4, and T6). (b) MRI of brain (T2 FLAIR): encephalomalacia in the right temporal region.
